# Genomes of *Endotrypanum monterogeii* from Panama and *Zelonia costaricensis* from Brazil: Expansion of Multigene Families in Leishmaniinae Parasites That Are Close Relatives of *Leishmania* spp.

**DOI:** 10.3390/pathogens12121409

**Published:** 2023-11-30

**Authors:** Percy O. Tullume-Vergara, Kelly Y. O. Caicedo, Jose F. C. Tantalean, Myrna G. Serrano, Gregory A. Buck, Marta M. G. Teixeira, Jeffrey J. Shaw, Joao M. P. Alves

**Affiliations:** 1 Department of Parasitology, Institute for Biomedical Sciences, University of Sao Paulo, Av. Prof. Lineu Prestes, 1374, Sao Paulo 05508-000, SP, Brazil; petullume@icb.usp.br (P.O.T.-V.); kellyolivos@usp.br (K.Y.O.C.); jcalderonta@ime.usp.br (J.F.C.T.); mmgteix@icb.usp.br (M.M.G.T.); jayusp@hotmail.com (J.J.S.); 2 Department of Microbiology and Immunology, Virginia Commonwealth University School of Medicine, 1101 E Marshall St., Richmond, VA 23298, USA; myrna.serrano@vcuhealth.org (M.G.S.); gregory.buck@vcuhealth.org (G.A.B.)

**Keywords:** *Endotrypanum monterogeii*, *Zelonia costaricensis*, sialidase, catalase, glycoprotein 63, phylogenomics, gene family expansion

## Abstract

The Leishmaniinae subfamily of the Trypanosomatidae contains both genus *Zelonia* (monoxenous) and *Endotrypanum* (dixenous). They are amongst the nearest known relatives of *Leishmania*, which comprises many human pathogens widespread in the developing world. These closely related lineages are models for the genomic biology of monoxenous and dixenous parasites. Herein, we used comparative genomics to identify the orthologous groups (OGs) shared among 26 Leishmaniinae species to investigate gene family expansion/contraction and applied two phylogenomic approaches to confirm relationships within the subfamily. The *Endotrypanum monterogeii* and *Zelonia costaricensis* genomes were assembled, with sizes of 29.9 Mb and 38.0 Mb and 9.711 and 12.201 predicted protein-coding genes, respectively. The genome of *E. monterogeii* displayed a higher number of multicopy cell surface protein families, including glycoprotein 63 and glycoprotein 46, compared to *Leishmania* spp. The genome of *Z. costaricensis* presents expansions of BT1 and amino acid transporters and proteins containing leucine-rich repeat domains, as well as a loss of ABC-type transporters. In total, 415 and 85 lineage-specific OGs were identified in *Z. costaricensis* and *E. monterogeii*. The evolutionary relationships within the subfamily were confirmed using the supermatrix (3384 protein-coding genes) and supertree methods. Overall, this study showed new expansions of multigene families in monoxenous and dixenous parasites of the subfamily Leishmaniinae.

## 1. Introduction

Trypanosomatids are a well-known and widely distributed group of obligatory flagellate parasites that infect invertebrates, vertebrates, and plants. During their long evolutionary history, these eukaryotic parasites adopted two kinds of life cycles. While most are monoxenous, infecting only one invertebrate host, a few groups are dixenous, being found in vertebrates/plants and invertebrates that are their vectors [1,2].

Presently, the family Trypanosomatidae is divided into 24 genera [3]. Recently, there have been substantial increases in the identification of new taxa, as well as changes in the taxonomy and nomenclature of the subfamily Leishmaniinae, leading to the creation of new genera and subgenera [4]. The dixenous genera are *Leishmania*, *Porcisia*, and *Endotrypanum*, and the predominantly monoxenous ones are *Zelonia*, *Crithidia*, *Leptomonas*, *Novymonas*, *Lotmaria*, and *Borovskyia,* which are parasites of Heteroptera, Diptera, and Hymenoptera [4,5,6]. An earlier study had split the genus *Leishmania* into the following two informal sections: Euleishmania and Paraleishmania [7]. According to a recent classification based on molecular phylogeny, the latter section comprises the species *Endotrypanum* and its closest relative, *Porcisia*. The genus *Endotrypanum* currently harbors five species *Endotrypanum schaudinni*, *Endotrypanum monterogeii*, and the species *Endotrypanum colombiensis*, *Endotrypanum equatorensis*, and *Endotrypanum herreri*, which were formerly classified as *Leishmania* [4].

According to the available data and the new classification of Leishmaniinae, human infection can be caused by *Leishmania* spp. and by *E. colombiensis,* which has been reported to cause both cutaneous and visceral leishmaniasis [7,8,9]. In addition to the increasing number of dixenous Leishmaniinae species infecting humans, molecular diagnosis has revealed concomitant infections of *Leishmania* species with *Leptomonas seymouri* and *Crithidia* spp. [10,11]. Evidence is accumulating that indicates that these monoxenous infections in man are related to a diminished immunity associated with other pathogenic parasites or an immunological deficiency. Therefore, the classification of trypanosomatids as monoxenous or heteroxenous must be viewed with caution. 

The distinctive feature of *E. schaudinni* and *E. monterogeii* is that they are the only known intraerythrocytic trypanosomatids of mammals; however, *Leishmania* spp. of the subgenus *Sauroleishmania* are also reported within lizard erythrocytes [12,13]. The xenarthrans *Choloepus didactylus* and *Choloepus hoffmanni* are the natural vertebrate hosts, and Phlebotominae sand flies are the vectors of *Endotrypanum* spp. [14,15,16]. 

Today, genomic studies with close relatives of the *Leishmania* emphasize the necessity of comparative studies since they share the same habitats in their invertebrate vectors as *Endotrypanum* [17,18,19]. The genus *Zelonia* was created by Shaw, Camargo, and Teixeira in 2016 to accommodate the species named *Leptomonas costaricensis* [4,20]. *Z. costaricensis* (IRIC/CR/2003/15EC) was isolated from the predator hemipteran *Ricolla simillina* (Heteroptera, Reduviidae) in Costa Rica in 2003, whereas our isolate of *Z. costaricensis* (IZEL/BR/89/169E) was isolated from the predator hemipteran *Zellus* sp. in Brazilian Amazonia [4,20]. At first, this species was based on the promastigote morphology and named *Leptomonas costaricensis*. However, in all analyses using small subunit and large subunit rRNAs and heat shock protein genes, this species was separated from *Leptomonas pyrrhocoris* and *Leptomonas seymouri* [20,21]. A previous phylogenetic study clustered *Z. costaricensis* with *Z. australiensis*, isolated in Australia from the black fly *Simulium (Morops) dycei* [21]. The phylogeny of the subfamily Leishmaniinae supports *Z. costaricensis* as a lineage basal to the clade harboring *Endotrypanum*, *Porcisia*, and *Leishmania* [4,21]. 

Cell surface proteins of trypanosomatid parasites have become important targets for comparative genomic studies. These protein families are considered to be of a cryptic nature due to their unknown origins in trypanosomatids [22,23,24]. The largest cell surface gene families have been identified in the genera *Trypanosoma*, *Leishmania*, *Crithidia*, and *Leptomonas* [25,26,27,28], including glycoprotein 63 (gp63), promastigote surface antigen (PSA, also known as gp46), amastin, tuzin, HASP (hydrophilic acylated surface protein), and SHERP (small hydrophilic ER-associated protein) [29,30,31,32]. 

The genome comparison of *Endotrypanum* and its closest known relative *Porcisia* (parasite of porcupines) revealed shared metabolic capacities with *Leishmania major* and a substantially reduced repertoire of surface proteins in both *Endotrypanum* and *Porcisia* compared to *Leishmania*, such as cell surface amastins required from the development of *Leishmania* amastigotes inside macrophages [33].

Comparative genomics approaches have been used to gain a better understanding of the genetic content in several trypanosomatids [22,24,34]. The focus of the present study is a genomic characterization of *E. monterogeii* from Panama (formerly named *E. schaudinni*) and *Z. costaricensis* from Brazil based on their whole-genome sequences and a comparative analysis with their close relatives, in particular those of the subfamily Leishmaniinae. This study also aims to confirm the phylogenetic position of *Z. costaricensis* and *E. monterogeii* using robust phylogenomics methods (supermatrix and supertree), employing 3384 single-copy proteins from 27 trypanosomatid lineages. 

## 2. Materials and Methods

### 2.1. Sample Origin, Genome Sequencing, and Assembly

High-quality genomic DNA was extracted, using the phenol–chloroform method, from *E. monterogeii* TCC224 (MCHO/PA/62/M907), which was originally isolated from sloth (*Choloepus hoffmanni*) in Colon Province, Panama, and from *Z. costaricensis* TCC169E (IZEL/BR/89/169E), which was isolated from a bug (*Zelus* sp.) in Amazonas, Brazil. These isolates are deposited in the Trypanosomatid Culture Collection of the University of Sao Paulo (TCC-USP).

Whole-genome sequencing was performed using standard pyrosequencing shotgun methodology on the Roche 454 platform, as recommended by the manufacturer. We estimated the quality of the sequences using FastQC tool v. 0.11.8 [35].

Genome assembly was performed using Roche’s Newbler software v. 2.3 (software distributed by the manufacturer). The pipeline Taxoblast v. 1.2 was used to identify and remove contigs from contaminants [36]. We calculated the N50 value using mfsizes v. 1.8.7 [37]. Additionally, to assess the completeness of the assemblies, Benchmarking Universal Single-Copy Ortholog (BUSCO) v. 5.2.2 [38] was run using the dataset Euglenozoa version 10 (n130 orthologous genes). Dot plot comparisons were run using the MUMmer tool v. 4.0 [39] to assess contig orientation and synteny. 

### 2.2. Gene Models, Repetitive Elements, and RNAs Prediction

To predict protein-coding genes, we employed GeneMarkS software v. 4.28 [40], using self-training mode, considering ORFs of at least 200 bp, and using the less intron parameter. In addition, we also submitted the genomes to AUGUSTUS web server v. 2 [41], using gene models from *L. tarentolae*. Prediction and annotation of non-coding genes was performed with tRNAscan-SE v. 1.4 [42] for tRNA annotation; RNAmmer v. 1.2.1 [43] to identify 5S, SSU, and LSU rRNAs sequences; and the INFERNAL package v. 1.1 [44] against Rfam database v. 12.0 [45] to identify snoRNA, and microRNA genes. Additionally, some gene models of interest were manually inspected to generate a final annotation dataset. 

For prediction of repetitive elements, RepeatModeler2 [46] pipeline was executed to construct a de novo repeat library for the *Z. costaricensis* and *E. monterogeii* genomes. The final library of each species was annotated with RepeatMasker software v. 4.1.2-p1 [47].

### 2.3. Functional Annotation of Genes 

We used a combination of public databases to assign functions to predicted gene models (proteins) in the *E. monterogeii* and *Z. costaricensis* genomes. We searched protein sequences against the non-redundant database from NCBI (downloaded 6 September 2021) [48], where we used BLASTP v. 2.9.0+, and BLASTN v. 2.9.0+ [49] with default parameters (except for the E-value cutoff of 1 × 10^−6^). We also submitted predicted proteins to EggNOG v. 5.0 database’s eggNOG-mapper v. 2.0 tool [50]. The proteomes were characterized functionally according to the eukaryotic orthologous groups (KOG) classification scheme [51]. As a complementary analysis, we used InterProScan v. 5.30-69.0 [52] in standalone mode with default parameters. Pfam [53], Gene Ontology, and InterPro domains database were employed to assign putative functions to the proteomes. The proteomes were also submitted to the KAAS website tool v. 2.1 [54] in the Kyoto Encyclopedia of Genes and Genomes (KEGG) database. 

### 2.4. Orthologous Group Analysis

Orthologous groups (OGs) were inferred using the OrthoVenn2 online tool [55] with default settings. Three species that have never been found infecting humans (*Porcisia hertigi* TCC260, *E. monterogeii LV88*, and *Z. costaricensis*) and one that is pathogenic to humans (*L. major* Friedlin) were selected for comparison of their protein data. *L. (L.) major* and *P. hertigi* were downloaded from TriTrypDB version 55 and 60 [56]. Also, specific-OGs for *Z. costaricensis* and *E. monterogeii* were selected for further analysis.

### 2.5. Datasets for Phylogenomic Analysis

Besides the two proteomes from this study, we downloaded protein-coding gene sequences from 25 taxa from TriTrypDB version 55 and the Genbank database (accessed on 1 October 2022) [56] (Table S1). The criterion for including a species was its membership in the subfamily Leishmaniinae, with the exception of *Blechomonas ayalai*, used as an outgroup. Single-copy orthologous groups as defined by Orthofinder2 v. 2.5.4 [57] were selected for the phylogenomic analysis. To evaluate the impact of missing data [58], we built two different datasets. Dataset 1 included all 3384 single-copy genes present in all 27 taxa, as defined above. Dataset 2 was built by selecting the 99 single-copy genes used by BUSCO, from all 27 species. Datasets were aligned by MUSCLE v. 3.8.31, [59], with subsequent removal of ambiguous aligned positions by Trimal v. 1.4.rev15, [60], using automated_1 parameters, the optimal method for trimming in phylogenetic analysis. The resulting filtered alignments from each dataset were concatenated into a corresponding supermatrix with FASconCAT-G v. 1.0 [61].

### 2.6. Phylogenomic Analysis of the Subfamily Leishmaniinae

Supermatrix and supertree approaches were used to infer phylogenetic relationships within the subfamily Leishmaniinae. The maximum-likelihood (ML) method was used for phylogenomic analyses based on the supermatrix (concatenated proteins) approach. Dataset 1 was analyzed with IQ-TREE2 v. 2.0.4 [62] using partitions for the individual proteins. The best substitution models were selected automatically by ModelFinder (MFP option) [63]. Branch support was measured with the aLTR algorithm [64]. Dataset 2 was analyzed without partitions, using the JTT model (Jones–Taylor–Thornton) [65] + Γ4 + F. To evaluate node support 1000 UFBS (Ultra-Fast Bootstrap) and 100 BS (non-parametric bootstrap) pseudoreplicates were run. Values higher than 95% and 75%, respectively, were considered robust. 

In addition to the ML method, we applied Bayesian inference (BI) to dataset 2, the smaller of the two. Dataset 1 was not analyzed using BI due to the large size of the supermatrix (3384 OGs), which made runtime impractical. The Bayesian tree was inferred using MrBayes v. 3.2.6 [66] with two independent Monte Carlo Markov chain (MCMC) analyses; each chain started from a random tree and was run for 10,000,000 generations after convergence, with sampling every 1000 generations. Node support was considered robust for posterior probabilities (PP) ≥ 0.95. 

The supertree approach was carried out with dataset 1, to verify for inconsistent results at the individual gene level, which might be associated with incomplete lineage sorting (ILS) [67]. For this, we estimated an individual gene tree for each of the 3384 single-copy genes with IQ-TREE2 using the JTT + Γ4 + F substitution model, and 1000 UFBS replicates to estimate node support. The supertree was inferred with ASTRAL-III v. 5.7.1 [68], and the option –t 1 was chosen to estimate quartet support. Trees were drawn with iTOL v. 5.4 [69] and cosmetic adjustments were performed in the Inkscape vector editor (www.inkscape.org, accessed on 1 October 2022). 

### 2.7. Characterization and Phylogenetic Analysis in gp63 Multigene Family

We selected gp63 (leishmanolysin), one of the gene families that have shown expansion, in *E. monterogeii* and 26 other genomes (Table S1). The selected gp63 proteins were used to find the Orthofinder orthologous groups of interest, which were then aligned with MUSCLE with default settings. The gp63 phylogenetic tree was inferred using IQ-Tree2. The best tree was chosen using the JTT + G4 model, and branch support was estimated by 100 bootstrap pseudoreplicates. Nodes were considered robust when displaying BS ≥ 0.75%. 

### 2.8. Metabolic Pathway Prediction 

We searched for potential biochemical pathways by deducing enzyme commission numbers and conducting comparative metabolic annotation analysis of four trypanosomatids (*L. (L.) major*, *E. monterogeii* LV88, *E. monterogeii* M907, and *Z. costaricensis*) using Asgard 1.6.5 [70], which uses Kyoto Encyclopedia of Genes and Genomes (KEGG) [71] and Uniref100 [72] databases as references. We then performed manual curation of the results of interest by thoroughly reading the relevant scientific literature [73,74]. To document significant enzymes in detail, we have used BRENDA [75]. Finally, the metabolic pathways of interest were then redrawn using Inkscape. 

## 3. Results

### 3.1. Assembly and Genome Characteristics

The final genome assembly size of *E. monterogeii* M907 was 29.66 Mbp, split into 10,088 contigs, presenting the largest contig with 41,500 bp and an N50 contig length of 6.0 kb. *Z. costaricensis* TCC169E had a genome assembly of 39.80 Mbp, with 7896 contigs, N50 of 17.4 kb and largest contig with 126,447 bp. The mean GC content was 52.66% and 64.26%, respectively. Assembly statistics are summarized and compared in Table 1. Additionally, dot plots were generated and displayed a high degree of synteny with other species of trypanosomatids, which indicates that, overall, the *E. monterogeii* and *Z. costaricensis* genomes are probably assembled correctly (Figure S1A,B).

To assess the completeness of genome assembly and gene model predictions, we ran a BUSCO analysis employing the Euglenozoa dataset from OrthoDB v.10 (130 BUSCO orthologs). The *E. monterogeii* M907 genome presented slightly more fragmentation (8 fragmented orthologs) than *Z. costaricensis* (2 fragmented orthologs). The genomes of high quality, such as *L. major* Friedlin, *L. pyrrhocoris* and *L. (V.) braziliensis,* all had no fragmented orthologs (Figure S2). To detect the presence of possible contaminants, we ran the taxoblast pipeline, detecting three prokaryote contigs on the draft assembly of *Z. costaricensis*, which were filtered out; the same analysis was performed in *E. monterogeii*, without any evidence of contaminants (bacteria).

### 3.2. Gene Prediction and Annotation

The best gene models were obtained using GeneMarkS. *E. monterogeii* and *Z. costaricensis* had 9711 and 12,201 putative protein-coding genes predicted, respectively, with a mean exon length of 1573 and 1580 bp, respectively. The content of repeat elements (RE) in the genomes varied, reaching 4.47% in *E. monterogeii* and 13.16% in *Z. costaricensis*. Intriguingly, in this analysis, no transposable elements (TEs) were found in *E. monterogeii*. In *Z. costaricensis*, various repeating elements, including those in multigene families, account for 4.21% of the genes, and unknown repeats are 2.43%. Among Tes, LTR, LINE, and SINE represent 0.28%, 0.10%, and 0.23%, respectively (Table S2). We found non-autonomous Ingi elements within LINE; additionally, LTR was composed of TATE (telomere-associated transposable element) and VIPER (vestigial interposed retroelement) elements.

Functional annotation in both species was carried out using the following multiple public databases: NCBI’s nr, KEGG, Pfam, EggNOG, and Interproscan (Table 2). Using BLASTP, 8834 *E. monterogeii* proteins had matches against the nr database (E-value cutoff of 1 × 10^−10^), which accounts for about 91% of the predicted proteins. Among those matches, 26% presented a high similarity to functionally annotated proteins, whereas 60.9% were assigned to hypothetical or unknown proteins. The remaining 13% of the proteome presented no hits against nr and were designated as unclassified or orphan. In addition, of the 8834 proteins, 8358 showed the best BLASTP hits with the genus *Leishmania* spp. and 387 with the genus *Leptomonas* spp.

KEGG mapping showed 2088 proteins (21.5%) in *E. monterogeii* and 2344 proteins (19.2%) in *Z. costaricensis* with matches against this database (Table 2).

**Figure 1:**
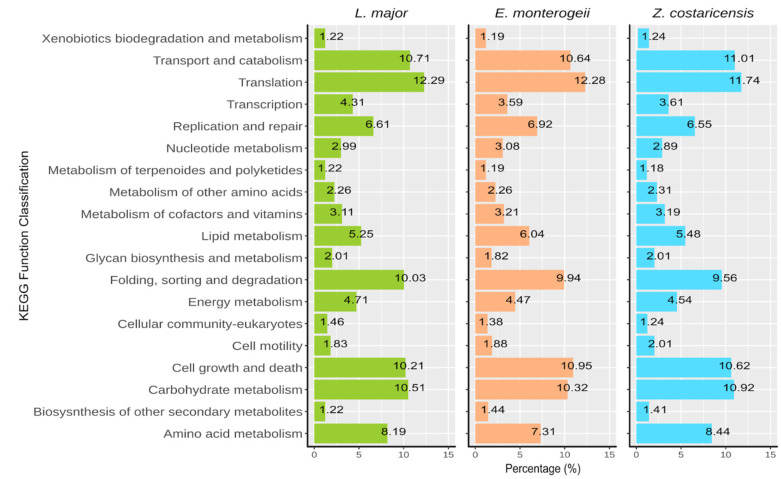
KEGG function classification for enzymatic characterization based in cellular processes, genetic information processing, and metabolic pathways using the *Leishmania (Leishmania) major*, *Endotrypanum monterogeii* and *Zelonia costaricensis* genomes.

Comparative analysis using three specific categories (cellular processes, genetic information processing, and metabolic pathway) are shown in Figure 1. Most genes were associated with pathways related to transport and catabolism, translation, folding, sorting and degradation, cell growth and death, and carbohydrate metabolism.

Furthermore, KOG assignments were used to assess the complexity of cellular functions in both species (Figure S3). The categories that had the highest numbers of genes were “Post-Transcriptional Modification and Protein Turnover, Chaperones”, “Signal Transduction Mechanisms”, “Translation, Ribosomal Structure and Biogenesis”, and “Carbohydrate Metabolism”.

**Figure 2:**
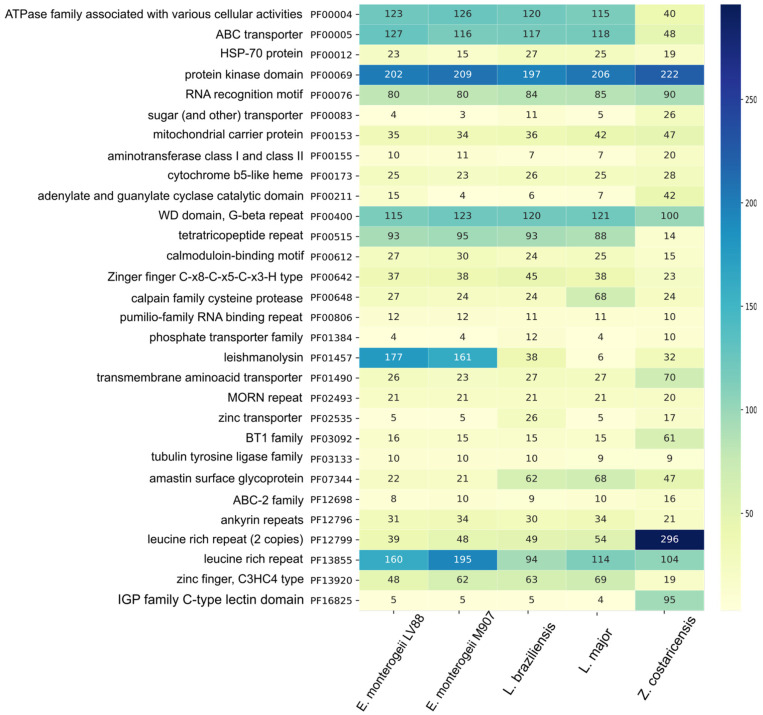
Abundance heatmap of 30 protein domain families in five Leishmaniinae species. The column on the left lists domain names along with Pfam code. The high number of domains is depicted in dark-blue and the low number of domains in light-green, following the color scale on the right.

Functional annotation based on Pfam domain families showed identified domains in 7241 (74.6%) and 8975 (73.5%) proteins from *E. monterogeii* and *Z. costaricensis*, respectively (Table 2). Additionally, a comparative analysis of Pfam domains using five lineages was carried out to establish the abundance of the domains among them. From these, we identified eight families that are more abundant in *Z. costaricensis* (Figure 2). These families include leucine-rich repeat (2 copies) (PF12799), IGP family C-type lectin (PF16825), BT1 (PF03092), amino acid transporters (PF01490), and sugar transporters (PF00083). We also noticed a lower abundance of domains (48 genes) associated with ABC-type transporter (PF00005), and tetratricopeptide repeat (PF00515). Conversely, the most abundant families in *E. monterogeii* were protein kinase (PF00012), leishmanolysin (PF01457), and leucine-rich repeat (PF13855). Interestingly, in *Endotrypanum* spp., the amastin multigene family (PF07344) showed low abundance (17 copies).

For the non-coding RNAs (Table 3), *Z. costaricensis* displayed 8 rRNAs, 201 tRNAs, 295 microRNAs, and 95 snoRNAs, while *E. monterogeii* had 6 rRNAs, 86 tRNAs, 307 microRNAs, and 213 snoRNAs.

### 3.3. Ortholog Clustering and Functional Analysis

**Figure 3:**
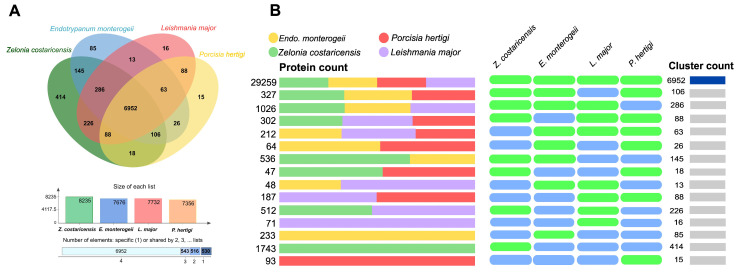
Comparative analysis of four species of Leishmaniinae. (**A**) Venn diagram of shared and unique orthologous group in four lineages. (**B**) Cluster count and proteins assigned to Ogs for *Leishmania (Leishmania) major*, *Endotrypanum monterogeii*, *Porcisia hertigi,* and *Zelonia costaricensis*. A core of 6952 orthologous groups was shared by all four lineages.

The annotated protein sequences from *Z. costaricensis* and *E. monterogeii* were also compared with other close relative parasites, such as pathogenic *L. major* and non-pathogenic *P. hertigi*, by orthologous group analysis. Of the 8541 orthologous groups (Ogs), 6511 were single-copy Ogs. There is a core of 6952 Ogs (with a total of 7506 proteins in *Z. costaricensis*, 7131 in *E. monterogeii*, 7339 in *L. major*, and 7265 in *P. hertigi*) that are shared by all four species (Figure 3A,B). Furthermore, the highest number of Ogs shared was that between *Z. costaricensis* and *L. major,* with 7552 orthologs, whereas *E. monterogeii* and *Z. costaricensis* shared 7489 Ogs (Table S3). Another 145 OG were shared between *Z. costaricensis* and *E. monterogeii* but not found in *L. major*. In total, 414 and 85 lineage-specific Ogs were identified in *Z. costaricensis* and *E. monterogeii*, respectively, which is higher than the numbers in *L. major* (16) and *P. hertigi* (15). Lineage-specific ortholog groups in *Z. costaricensis* were involved in the plasma membrane, pathogenesis, intracellular signal transduction, glycerol transport, and cellulose catabolic process. In *E. monterogeii*, they were implicated in cell adhesion, phospholipid translocation, and intracellular signal transduction. In addition, both species showed several Ogs that were hypothetical proteins.

### 3.4. Phylogenomics Analysis into the Subfamily Leishmaniinae

The evolutionary and phylogenetic relationships of the *E. monterogeii* and *Z. costaricensis* species that belong to the subfamily Leishmaniinae were inferred by applying the supertree and supermatrix phylogenomic approaches. Two datasets were created based on single-copy genes. In the largest matrix, named Dataset.1 3384 single-copy genes were concatenated representing 50,950,701 amino acids, 1,198,087 indels, 49,752,083 parsimony-informative sites, and 531 missing data. Conversely, the smallest matrix, Dataset.2, was obtained from 99 single-copy genes (conservative) from the BUSCO analysis, which represented 2,209,302 amino acids, 38,781 indels, and 2,170,511 parsimony-informative sites.

**Figure 4:**
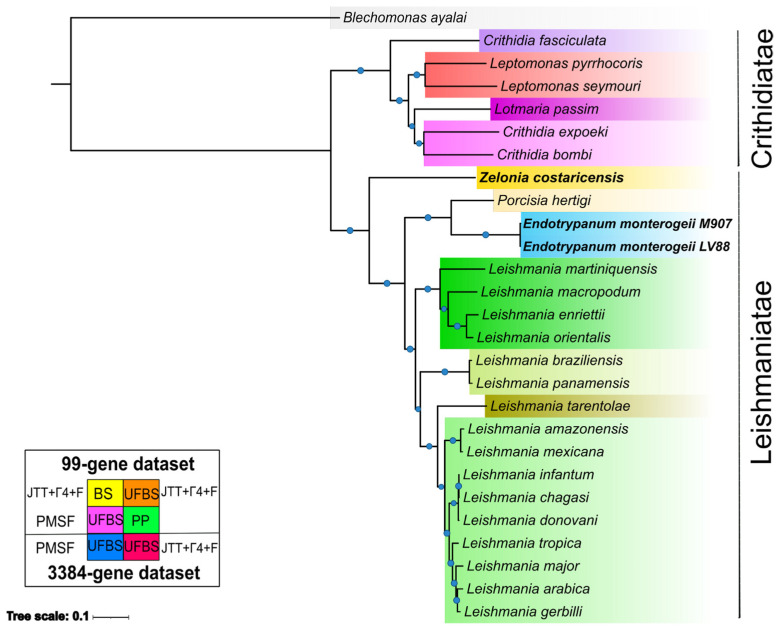
Maximum likelihood (ML) phylogenomic tree based on a concatenated dataset of 3384 single-copy genes. The tree was reconstructed using 27 species, and 5 different models using ML and 1 model using BI were tested. All analyses recovered the same topological structure. UFBS, BS, and PP values higher than 95%, 75%, and 1 are shown as light blue circles. The horizontal bar depicts 0.01 substitutions per site.

The phylogenomic tree was generated based on a group of 27 taxa. In brief, we applied several methods, statistical support metrics, and two datasets in order to find variation on the topological structure of species tree (Figure 4 and Figure S4A–E, Table S4). All of the trees recovered showed that the subfamily Leishmaniinae was split in two known clades, the infrafamily Leishmaniatae, which is a group where almost all members are dixenous, and the infrafamily Crithidiatae, which harbors monoxenous species. As expected, *Z. costaricensis* is placed in a basal branch into the Leishmaniatae, composed of *P. hertigi*, *Endotrypanum* spp., and *Leishmania* spp., with high support (BS: 100, PP: 1).

The monophyletic *Leishmania* clade was split in its expected subgenera, with *Mundinia* located basally, followed by *Viannia*, then *Sauroleishmania*, and *Leishmania*. The *Endotrypanum* + *Porcisia* clade was recovered as the outgroup for the genus *Leishmania*, and also showed strong support (BS:100, PP: 1). Additionally, *E. monterogeii* M907 and *E. monterogeii* LV88 are placed together and with negligible branch lengths, indicating the very low levels of sequence divergence between the two strains.

To confirm that topological variation among different gene phylogenies was not interfering with the overall phylogenetic signal that led to the supermatrix tree, we also ran a supertree analysis using ASTRAL to infer the separate history of the 3,384 single-copy genes. The phylogenomic tree obtained had the same topology as the supermatrix method, and high support for the nodes (LPP = 1) (Figure S5); moreover, we estimated the quartet score to be 53,851,596 and the normalized quartet score to be 0.919019. This means that 53,851,596 induced quartet trees from the gene trees are in the species tree, and these are 91.90% of all quartet trees that can be found in the species tree.

### 3.5. Phylogenetic Analysis of the GP63 Family

GP63 belongs to a multigene family and its functions have been extensively studied in *Leishmania* spp. [29]. For example, gp63 plays a role in the interaction and internalization with the macrophage [76,77]. Previous studies revealed a significant expansion of the gp63 family on chromosome 10 in *L. (V.) braziliensis* and *L. (Sauroleishmania) tarentolae* [78,79].

This expansion also happened in the genus *Endotrypanum* sp. (Table S5). We performed an evolutionary analysis of the gp63 family of *E. monterogeii* and *Z. costaricensis*. The 161 gp63 sequences identified and annotated in *E. monterogeii* were distributed in 28 ortholog groups, the largest being the following: OG00000 (with 69 gp63 genes) and OG00011 (with 52 gp63 genes) (Table S5). The length of the sequences ranged from 99 to 899 amino acids; the most frequent sequence length in *E. monterogeii* ranged from 100 to 199 aa (Figure S5). We also identified conserved motifs within gp63 sequences (Figure S6). For clarity, only the two most numerous groups (406 and 142 protein sequences) were considered when building these trees (Table S7).

**Figure 5:**
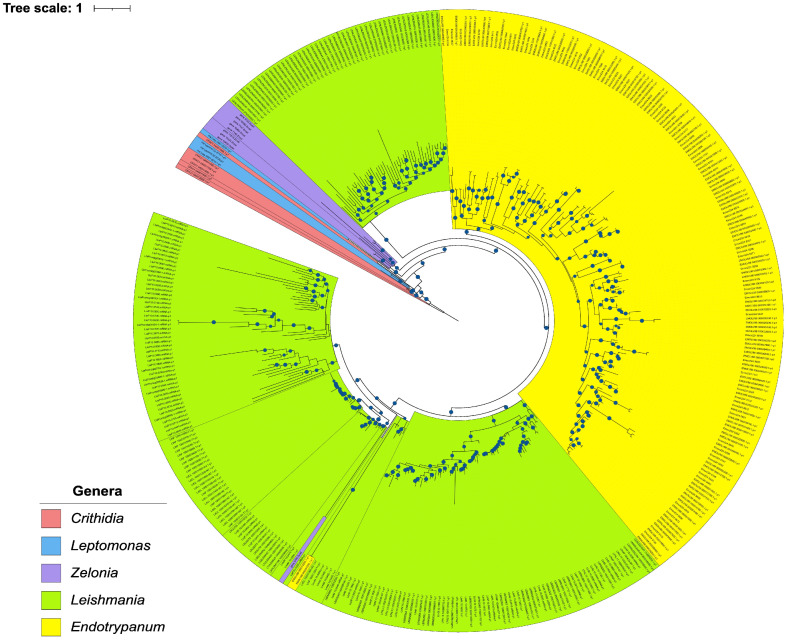
Phylogenetic relationships of glycoprotein 63 in 21 species using the ML method. The phylogram is represented by 406 gp63 protein sequences. The sequences from *Zelonia* and *Endotrypanum* genera are shown in red and yellow, respectively. Bootstrap exceeding 95% are shown in branches as blue circles.

The gp63 phylogenetic tree (Figure 5) mostly reflected species phylogeny. The basal clades belong to the genera *Crithidia* and *Leptomonas,* as expected. Most *Zelonia* gp63 sequences were placed external to the *Leishmania* and *Endotrypanum* genera. Even though *Endotrypanum* is a genus that diverged early in comparison with *Leishmania*, an interesting set of *Leishmania* spp. Gp63 sequences have possibly diverged in a former event.

### 3.6. Metabolic Pathway Comparison between L. (L.) major, Z. costaricensis, and E. monterogeii

By using comparative analysis with the Asgard tool, we screened 385 modules (metabolic pathways) from KEEG, searching for similarities and differences among all four taxa (*E. monterogeii* M907, *E. monterogeii* LV88, *Z. costaricensis*, and *L. major*). All pathways at the enzymatic level were almost identical for the three genera. We show some of the most distinctive enzymes differentially found in these three genera in Tables S6 and S7.

**Figure 6:**
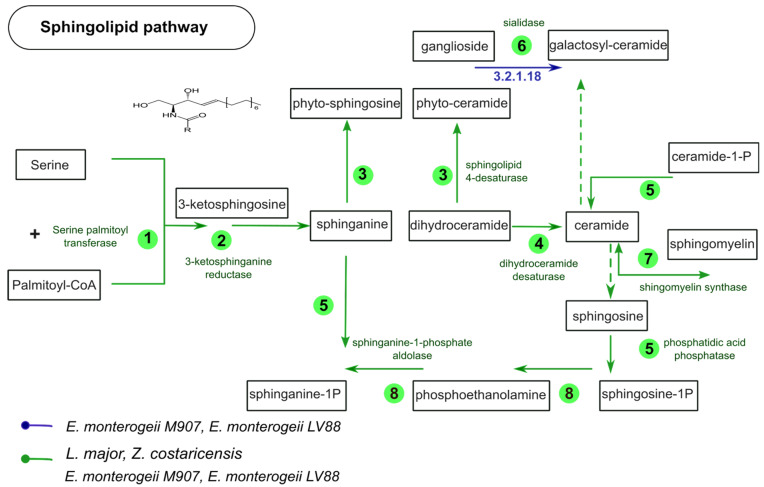
Schematic representation of sphingolipid metabolism comparing four *Leishmaniinae* parasites. The numbers in green circles represent the number of predicted enzymes that are present in each pathway and in the four lineages. The blue arrow shows the sialidase enzyme present only in *Endotrypanum monterogeii* strains but absent in *Leishmania (Leishmania) major*.

We found ten *Zelonia*-specific enzymes, which include catalase (E.C. 1.11.1.6), penicillin acylase (E.C. 3.5.1.11), and argininosuccinate lyase (E.C. 4.3.2.1). Likewise, we also looked for specific enzymes in *Endotrypanum*, finding only two, sialidase (E.C. 3.2.1.18) and phosphoglucosamine mutase (E.C. 5.4.2.10). Based on these, we inferred the sphingolipid pathway, as defined by KEGG, comparing the four species (Figure 6). Seven of the enzymes were conserved in all of the species, with sialidase as the only one exclusive to *E. monterogeii*.

A comparative enzymatic analysis between *Leishmania* and *Endotrypanum* revealed 485 KEGG ortholog enzymes. Furthermore, this analysis identified 13 specific enzymes of *Leishmania* that were absent in *Endotrypanum* spp.

Trypanosomatids are unable to synthesize heme, however, the last three enzymes of heme biosynthesis were acquired from bacteria by horizontal gene transfer (HGT) [80]. Alves et al. 2011 have identified these three genes in some Leishmaniinae, such as *Leishmania*, *Zelonia* (at the time, classified as *Leptomonas*), *Angomonas*, *Strigomonas*, *Crithidia*, and *Endotrypanum* [81]. Accordingly, we also screened the *Z. costaricensis* genome identifying these last three coding genes (PPOX, CPOX, and FeCH) (Table S7).

## 4. Discussion

Since the establishment of the subfamily Leishmaniinae by Jirků in 2012 [82], molecular phylogenetics studies have improved the current taxonomic organization of the Leishmaniinae parasites. This includes the setting up of two new genera, *Zelonia* and *Porcisia*, and the new subgenus *Mundinia*; it was recognized that some species previously considered as belonging to the genus *Leishmania* were, in fact, species of *Endotrypanum* [4,8]. Expanding the genomic biology of the basal Leishmaniinae lineages [17] will potentially help in elucidating the monoxenous transition from a monoxenous to a dixenous life cycle. Until the present study, only a few genomes of monoxenous Leishmaniinae have been published [27,28,33,83]. In this study, we performed the first large-scale genomic analysis of a member of the genus *Zelonia*, a Brazilian isolate of *Z. costaricensis*, and compared it to that of the dixenous parasite *E. monterogeii* and other closely related dixenous and monoxenous Leishmaniinae. The idea is that these comparisons will highlight the expansion or contraction of different gene families that contribute to different life cycles.

The *E. monterogeii* M907 genome size (~29.9 Mb) was close to that of *E. monterogeii* LV88 (30.4 Mb), and also similar in size to that of *P. hertigi* (~29.1 Mb) and *L. major* (32.8 Mb). [26,33] (Table 1). Interestingly, *Z. costaricensis* was assembled in ~38.8 Mb, a size that is close to that of *C. fasciculata* (~41.2 Mb). We postulate that this increase in genome size can be attributed to the occurrence of duplication events, explicitly, the expansion of multigene families, which comprise approximately 4.21% of the genome of *Z. costaricensis* (Table S2). In trypanosomatids, repetitive content can reach 50% in *Trypanosoma cruzi* and 10% in *L. major*, of which 29% and 4% are multigene families, respectively [22,26,84]. On the other hand, *Z. costaricensis* and *E. monterogeii* have 12,201 and 9,711 predicted protein-coding genes, respectively, similar to other Leishmaniinae spp., such as *C. fasciculata* CfCl, *Lotmaria passim,* and *L. pyrrhocoris* H10 [6,27,28].

Earlier phylogenies suggest that *Z. costaricensis* is placed in an early branch of the Leishmaniinae tree, basal to the *Endotrypanum*, *Porcisia*, and *Leishmania*. Given that these phylogenies were built using few genetic markers [4,21,85], this study employed a more robust phylogenomic analysis using whole-genome sequences to confirm the placement of *Z. costaricensis* TCC169E and *E. monterogeii* M907 (previously referred to as *E. schaudinni* by Mesnil and Brimont, 1908). Despite the relatively fragmented nature of our genomes (Table 1), we obtained a very well supported and fully resolved phylogeny by adopting phylogenomic methods.

The phylogenomic tree was inferred with a variety of methods (ML and IB), statistical support metrics (BS = 100% and UFBS = 100%), and two different datasets (99 vs. 3384 protein-coding genes) in order to find variation in the topologies of the species tree. *B. ayalai*, a parasite of fleas (Siphonaptera) [86], was used as an outgroup. Additionally, due to the fact that spurious high support values (100% = UFBS, 100% = BS, and 1 = PP) can be seen in all nodes of phylogenomic trees using the supermatrix method, we also performed our inference using the supertree approach (Figure S5).

In all methods employed, we recovered *Z. costaricensis* as the sister group of *Porcisia*/*Endotrypanum* and *Leishmania*, in the infrafamily Leishmaniatae, in agreement with previous studies [33,85]. The genus *Endotrypanum*, along with the genus *Porcisia*, has previously been found to be an early branch compared to *Leishmania* [4,33]. In agreement with previous studies using different methods, our analyses confirmed, using ML, Bayesian inference, and quartet-based methods, the robust phylogenetic placement of the genus *Endotrypanum* spp. [4,7,33]. The two *E. monterogeii* strains are located on the same branch and with branch lengths of nearly zero, confirming the findings of Espinosa et al. (2016) showing that the formerly named *E. schaudinni* M907 isolate is actually the same species as *E. monterogeii* LV88 (MCHO/CR/62/A9).

The two phylogenomic trees obtained in this work fully agree with previous analyses (e.g., [4,5]) based on classical markers for trypanosomatids such as the SSU rRNA gene, the ITS rDNA, and the glycosomal glyceraldehyde 3-phosphate dehydrogenase (gGAPDH) gene. However, it is worth noting that these markers have different resolutions that are suitable for specific situations. Within the Leishmaniinae, primary sequence evolution is sometimes slow enough (e.g., within the *Viannia* subgenus of *Leishmania*) that only the ITS rDNA of the three classic markers has enough phylogenetic signal to separate closely related species in the subfamily. Our phylogenomic analyses, employing 99 or 3384 genes displaying a broad range of evolutionary rates, confirm that the classic markers continue to be valuable tools in studies involving large numbers of isolates, for which genome sequencing is not efficient or not of interest. On the other hand, the use of many single-copy protein sequences yields phylogenies with much greater statistical support while also practically eliminating the risk of using markers whose gene trees differ from the species tree (even though that is an uncommon problem for the three classic markers used for trypanosomatid phylogeny, it remains a possibility). Therefore, marker selection should be performed on a case-by-case basis, depending on the specifics of each study.

Gene duplication is an essential mechanism that drives genome evolution, being a factor responsible for the expansion of gene families [87]. The trypanosomatid genomes are characterized by exhibiting a tandem duplication mechanism and, through this event, they have extended the number of multigene families [24]. For instance, cation transporter genes in *Trypanosoma* spp., and amino acids transporter (AAT), receptor-type adenylate cyclase, and gp63 genes in *Leishmania* spp. have been seen forming tandem gene arrays [24,88].

Gp63 proteins are zinc-dependent metalloproteases that form the structural basis of the parasite’s cellular membrane. In *Leishmania*, gp63 plays important roles in host cell entry, immune modulation, intracellular survival, tissue invasion, and antigenic variation, which make it a key virulence factor in *Leishmania* infection. It inactivates the complement cascade, preventing damage to the parasite’s membrane while allowing opsonization and phagocytosis. By facilitating parasite binding to macrophages through fibronectin receptors, gp63 reduces the production of TNF, IL-12, and nitric oxide, further promoting parasite survival. It can degrade host cell proteins, promoting the acquisition of nutrients from the host cell, and contribute to the establishment of the parasitophorous vacuole, the specialized compartment that guarantees the parasite’s intracellular survival. Gp63 also activates a host tyrosine phosphatase, which aids in the parasite’s entry into macrophages [29,76,77].

In this study, 161 gp63 genes were identified and annotated in the *E. monterogeii* M907 genome. This is a significant increase compared to *Z. costaricensis* with 30 gp63 genes. To verify this high number, we screened the *E. montegoreii* LV88 genome from TriTrypDB (Table S1), and also found 171 sequences annotated as gp63 or leishmanolysin (Figure 3). Additionally, our phylogenetic analysis of gp63 (Figure 5) suggests that the expansion of this multigene family seen in *Endotrypanum* spp. may have happened after its split from the *Leishmania* genus.

Like *Endotrypanum*, *T. cruzi* possesses a large gp63 family (Tcgp63), with 60 proteins split into subfamilies; the Tcgp63-I and II groups have shown features of metalloproteases, while Tcgp63-III may contain some pseudogenes [89]. In *L. (V.) braziliensis*, 38 gp63 genes have been identified, of which the ones on chromosome 10 genes are associated with parasite interaction with the mammalian host, while another gp63 group is related to adhesion in the insect gut epithelium [78,90]. Pereira et al. (2009) reported that gp63 genes were involved in the interaction of *Leptomonas* spp. with their insect vector [91].

The large gp63 repertoire found in *Endotrypanum* opens a new focus of investigations on the evolution of the digenetic life mode. Its relatively low genetic expression in the monogenetic *Zelonia* suggests that increasing the number of gp63 genes could have been a crucial evolutionary step in the transition from the monogenetic to the digenetic lifestyle. We hypothesize that the gp63 multigene family may be intimately linked to novel features reflecting the adaptation to the digenetic life cycle within the Leishmaniinae.

Also, the expansion of multigene families has been a fundamental step for parasites to adapt and survive inside their host [24]. In this study, we identified 70 amino acid transporter (AAT) genes in *Z. costaricensis*, higher than in *Leishmania* spp., as shown in Figure 2. The AAT gene family has been biochemically well-characterized in a few trypanosomatids. Some AATs perform the uptake of L-arginine in *L. (Leishmania) amazonensis* and *L. (L.) donovani*, proline in *L. (L.) donovani* and *T. cruzi*, and glutamate in *T. cruzi* [92,93,94]. Mazared et al. (1999) identified that the activity of proline transporter has been active in the promastigote stage (insect host) in *L. (L.) donovani* [95]. Moreover, the duplication events are induced and presumably maintained to meet the requirements of specific life cycle stages or to regulate amino acid uptake, as occurred in *T. cruzi*, *T. brucei,* and *L. (L.) major* [88].

Gene repertoires could be reduced due to adaptation of parasitic lifestyle into vertebrates or plants, as happened in *Leishmania* and *Phytomonas* [73,96]. Thus, we can explain that this large number of ATT genes is essential to adapt to its way of life inside the insect host, in which the parasite captures amino acids as their main source of energy. Indeed, the parasites inside the insect have access to a number of amino acid sources obtained from the insect’s hemolymph, as well as amino acids from bacterial sources [97].

Conversely, ATP-binding cassette (ABC)-type transporters are another multigene family that displayed a decrease in the number of copies in *Z. costaricensis*. ABC comprises one of the largest protein families in living organisms, having conserved ABC domains that bind to and hydrolyze ATP [98]. In *Leishmania* spp., ABC transporters have been well-characterized into eight subfamilies (42 genes) and were sometimes implicated in drug resistance mechanisms [99]. For example, there is evidence that the ABC transporter MRPA subfamily confers antimony resistance [100]. Another, ABCC7 transporter, was a candidate to confer resistance to pentamidine in the amastigote and promastigote stages in *L. major* and *L. (L.) amazonensis* [101].

In our analyses of *Zelonia*, we identified 40 ABC-type transporter proteins, which is a low number when compared to *L. (L.) major* and *E. monterogeii*, with 120 and 126 proteins, respectively (Figure 2). Probably, this monoxenous organism did not need to increase its arsenal of ABC transporters, as probably occurred in *Leishmania* spp. In agreement with our data, this pattern of reduction in ABC-type transporter genes has already been reported in the genome of *L. pyrrhocoris* [28], suggesting that the low repertory of such genes might be a characteristic for its lifestyle in the insect.

Trypanosomatid metabolism has evolved to adapt to new environments and food supplies in vertebrate and invertebrate hosts [102]. The evolutionary events of gain and loss of metabolism-related genes can be used to infer switches in the lifestyles of monoxenous and dixenous parasites [97]. Interestingly, Leishmaniinae is the only trypanosomatid subfamily with a notable gain of metabolic genes (23 enzymes), possibly of a bacterial origin [3]. *Leishmania* spp. contains within its genome a complex metabolic machinery, which allows alternations between insect vectors and vertebrate hosts [103,104]. We screened for enzyme-coding genes by comparing four lineages of Leishmaniinae and have seen that most enzymes are present in all four with a few exceptions (Table S7). Then, we focused on two interesting genes, one specific to *Endotrypanum* spp. and the other to *Z. costarincensis*.

Trans-sialidase is a large multigene family characterized in both *T. cruzi* and *T. brucei* [22,25,105]. Over ~1400 genes have been arranged in eight subfamilies, with special interest in the type I subfamily, which is associated with trans-sialidase/neuraminidase enzymatic activities [106]. Trans-sialidase has been localized in the cell body, flagellum, and flagellar pocket [107,108]. *Endotrypanum*, a lineage distantly related to *Trypanosoma*, has also shown high sialidase activity [109]. Herein, we confirmed an *Endotrypanum*-specific gene, which might be used in the sphingolipid pathway (Figure 6 and Table S7). Interestingly, this sialidase shares orthologs in both *T. cruzi* and *T. brucei*, suggesting that it may be a vestigial gene that still plays an important role in *Endotrypanum* spp.

The main function of sialidase is to transfer sialic acid from one glycoconjugate to another [110]. Another enzymatic activity of sialidase observed during the trypomastigote stage of *T. cruzi* is that, when released into the extracellular environment, it can be dispersed into the blood [111]. *T. cruzi* is unable to synthesize sialic acid, but it has a sialidase receptor to acquire sialic acid units from mammalian host glycoconjugates [112,113]. That way, at the trypomastigote stage, *T. cruzi* has a molecular tool to protect itself from host complement, enabling its survival in the bloodstream [114,115]. Given this ability of *T. cruzi*, it is possible that the sialidase enzyme of *Endotrypanum* spp. exerts essential enzymatic activity to evade attack via the vertebrate host’s immune system, more specifically, attack via the complement system. Furthermore, we believe that sialidase can be used as an *Endotrypanum*-specific marker enzyme to aid in epidemiological identification, especially when doing insect host surveys [109].

Previous studies have emphasized the critical role of the catalase enzyme, deeming it essential for the monoxenous lifestyle; accordingly, it has been identified in *Novymonas*, *Leptomonas,* and *Crithidia* genera, but it has been lost by *Leishmania* spp., presumably due to its adaptation to a dixenous lifestyle [116,117]. The enzyme catalase has been found only in the subfamily Leishmaniinae, and it was acquired through a recent HGT event [117]. *Leptomonas*, *Crithidia,* and *Novymonas* probably acquired their catalase enzyme from a *Brachyspira* spp. (Spirochaetes) bacterium, while *Blastocrithidia* spp. might have acquired its catalase gene from a bacterium related to *Snodgrassella alvi* (Betaproteobacteria). The catalase enzyme identified in *Zelonia* spp. (Table S7), has displayed 85% and 75% identities with the orthologs from *Novymonas esmeralda* and *B. alvinipulli*, respectively (data not shown). This result suggests that the *Zelonia* catalase is derived from a common ancestor with *N. esmeralda*, which has been strongly supported by previous studies [117,118].

Host-parasite interaction mechanisms appear to be conserved in trypanosomatid parasites, although there are some variations depending on the proteomic machinery of the organism [119]. Interestingly, the host immune system became the primary driving force behind trypanosomatid evolution, constantly placing the parasite’s surface under selective pressure [120]. Consequently, surface protein families have evolved to enable trypanosomatid parasites to cope with environmental stresses encountered during their extracellular stage in the invertebrate host or intracellular stage in the vertebrate host [121]. Previous phylogenetic studies have demonstrated the relationship between subfamilies of surface proteins and their association with monoxenous or dixenous life cycles [121]. For instance, amastin has been categorized into four distinct subfamilies (α, β, γ, and δ) [96]. The position of basal amastins from the free-living kinetoplastid *Bodo saltans* is closely related to the α-amastin subfamily. Conversely, in the dixenous *Leishmania*, an expansion of the δ-amastin subfamily is associated with macrophage-parasite interaction. This evolutionary event coincides with the transition to the intracellular stage.

The comparative approach used in this study clearly suggests the existence of expanded multigene families that might confer adaptations to their life cycle, which alternates between insect and vertebrate host. Lastly, although short read-based sequencing technology produced fragmented genome assemblies, this did not affect the analysis and annotation of the whole genome sequences of *Z. costaricensis* and *E. monterogeii* (Figure S2 and Table 1). Nevertheless, based on the new findings about repetitive gene families uncovered in this study, we strongly believe that these genomes should be newly sequenced using third-generation sequencing [23], which should avoid assembly artifacts that collapse such repetitive families and thus more confidently characterize the significant expansion of multigene families in both genera.

## 5. Conclusions

The present comparative genomics study provides new insights into the possible mechanisms of host adaptation of *Zelonia costaricensis* and *Endotrypanum monterogeii*. It highlights the lineage-specific expansion in *E. monterogeii* of important gene families that interact with their host, such as gp63 and gp46, which are important modulators of *Leishmania* species. In *Z. costaricensis*, a large number of multigene families are involved in biopterin, amino acid transporters, adenylate cyclase, and hypothetical proteins carrying leucine-rich repeats (2 copies) and C-type lectin domains of the IGP family. A phylogenomic analysis using robust methods (ML and IB) and approaches (supermatrix and supertree) of single-copy genes confirmed the position of the two genera within the Leishmaniinae. The presented data add to our understanding of the genome biology of *Zelonia* (monoxenous) and *Endotrypanum* (dixenous), close relatives of *Leishmania* species, and the expansion and retraction of multigene families in trypanosomatids. Future genomic studies of other monoxenous Leishmaniinae will help in understanding the evolutionary acquisition of the dixenous life cycle.

## Figures and Tables

**Table 1:** Assembly statistics and completeness assessment for the *Endotrypanum monterogeii* M907 and *Zelonia costaricensis* TCC169E genomes.

Assembly Features	*E. monterogeii* M907	*Z. costaricensis* TCC169E
Number of contigs	10,088	7896
Longest contigs (bp)	41,459	126,447
N50 length (bp)	6007	17,448
L50	1445	614
GC%	52.66	64.26
Total length (Mb)	29.66	38.8
BUSCO v. 5.2.2		
Complete single-copy	125	129
Complete single duplicated	0	0
Fragmented	5	1
Missing	0	0

**Table 2:** Comparative genomics metric for *Endotrypanum monterogeii* and *Zelonia costaricensis*.

Features	*E. monterogeii*	Percentage	*Z. costaricensis*	Percentage
Protein-coding genes	9711	NA	12,201	NA
G + C% of CDS	57.12	NA	68.39	NA
Mean exon length (bp)	1580	NA	1573	NA
Nr-DB *	8834	90.90%	11,380	93.30%
KAAS-KEGG *	2088	21.50%	2344	19.20%
Pfam-DB *	7241	74.60%	8975	73.50%
EggNOG-DB *	7738	79.60%	9173	75.20%
Interproscan *	8497	87.50%	11,171	95.50%

*: indicates number of proteins and percentage of whole proteome matching the database; NA: not applicable.

**Table 3:** Non-coding RNA prediction summary.

Types	*Z. costaricensis*	*E. monterogeii*	Software
rRNA	8	6	RNAmmer
tRNA	201	295	tRNAscan
microRNA	295	307	Infernal
snoRNA	95	213	Infernal

## Data Availability

The assembled genomes generated in this work are available from NCBI under accession numbers JAXHDX000000000 (Zelonia costaricensis IZEL/BR/89/169E) and JAXHDY000000000 (Endotrypanum monterogeii MCHO/PA/62/M907).

## Supplementary Materials

The following supporting information can be downloaded at: https://www.mdpi.com/article/10.3390/pathogens12121409/s1, Figure S1: Dot-plots representing whole genome comparison.; Figure S2: Genomic quality assessment of the 27 trypanosomatids genomes used in this study.; Figure S3: Histogram of functional classification of the genomes of the three species Leishmaniinae according to KOG functional groups.; Figure S4: (a) Phylogenomics analysis of subfamily Leishmaniinae based on the supermatrix method using 99 BUSCO conservative genes (JTT + Γ4 + F + BS). (b) Phylogenomics analysis of subfamily Leishmaniinae based on the supermatrix method using 99 BUSCO conservative genes (JTT + Γ4 + F + UFBS). (c) Phylogenomics relationships of subfamily Leishmaniinae based on the supermatrix method using 99 BUSCO conservative genes (PMSF+UFBS). (d) Phylogenomics relationships of subfamily Leishmaniinae based on the supermatrix method using 99 BUSCO conservative genes (inference Bayesian). (e) Phylogenomics relationships of subfamily Leishmaniinae based on supermatrix analyses (JTT + Γ4 +F + UFBS) on 3384 single-copy genes.; Figure S5: Phylogenomics relationships of subfamily Leishmaniinae based on supertree analyses.; Figure S6: A bar graph indicating the comparison of the number of sequences between *E. monterogeii* LV88 (from TritrypDB) and *E. monterogeii* M907.; Figure S7: GP63 conserved motif in *E. monterogeii* M907. [28]; Table S1: List of genomes sequenced in this work and several available 25 trypanosomatid genome assemblies. [6,27,28,33,34,83,121,122,123,124,125,126,127,128,129,130,131,132,133]; Table S2: Percentage of repetitive element categories representation in the genome of *Z. costaricensis* and *E. monterogeii*.; Table S3: Ortholog clusters analysis using OrthoVenn version 2 webtool.; Table S4: Methods and models used in this phylogenomics analysis.; Table S5: Number of gp63 proteins forming ortholog groups in *Endotrypanum monterogeii*.; Table S6: List of specific enzymes for *Leishmania major*; Table S7: Summary of specific enzymes for *Zelonia costaricensis*, *Endotrypanum monterogeii*, and specific between *Z. costaricensis* and *Leishmania major*.
