# Supplementary material for: Genomes of Endotrypanum monterogeii from Panama and Zelonia costaricensis from Brazil: Expansion of Multigene Families in Leishmaniinae Parasites That Are Close Relatives of Leishmania spp
Source: Pathogens. 2023 Nov 30;12(12):1409. doi: 10.3390/pathogens12121409 (PMC10747355; doi:10.3390/pathogens12121409)
Supplement: Supplementary file 1 [file pathogens-12-01409-s001.zip › Suppl_Fig_Endotrypanum.pdf]

Supplementary Material

A

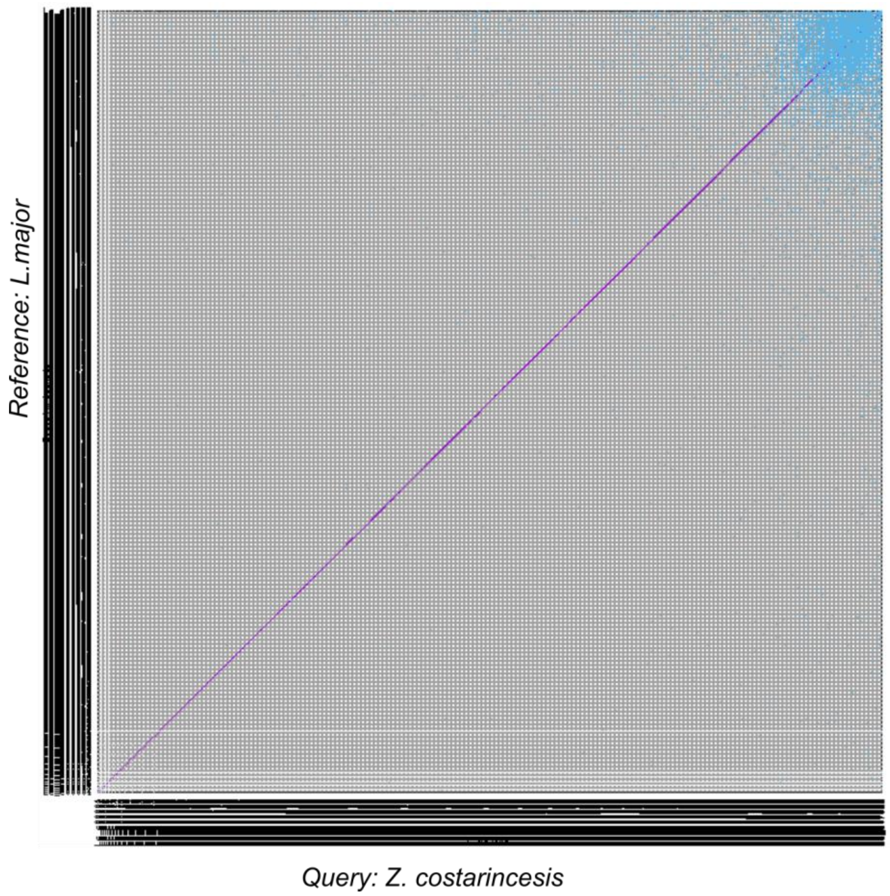

B

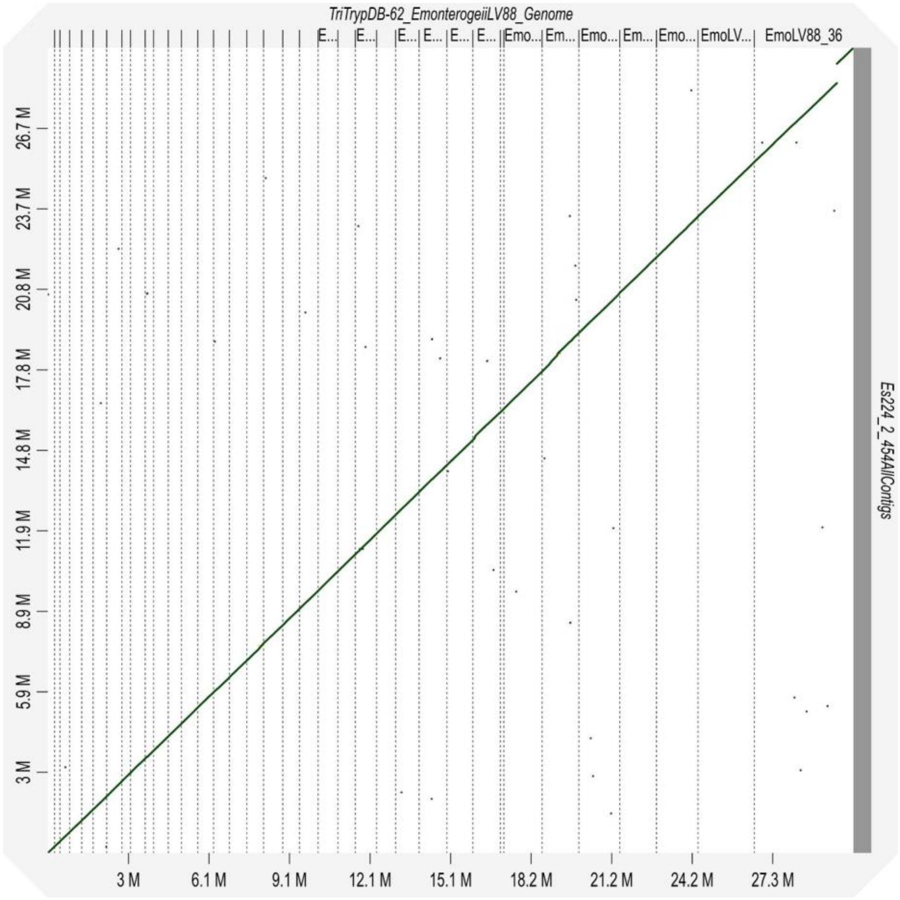

FigureS1. Dot-plots representing whole genome comparison. A. The comparison between *L. major* Friedlin and *Z. costaricensis* strain 169. This dot-plot was performed using MUMmer (NUCmer). B. Another comparison between *E. monterogeii* M907 and *E. monterogeii*LV88 using DGenies webtool. The dots or diagonal line (purple line [>=85%] and green line [>=95%]) depicts the high sequence identity between both genomes.

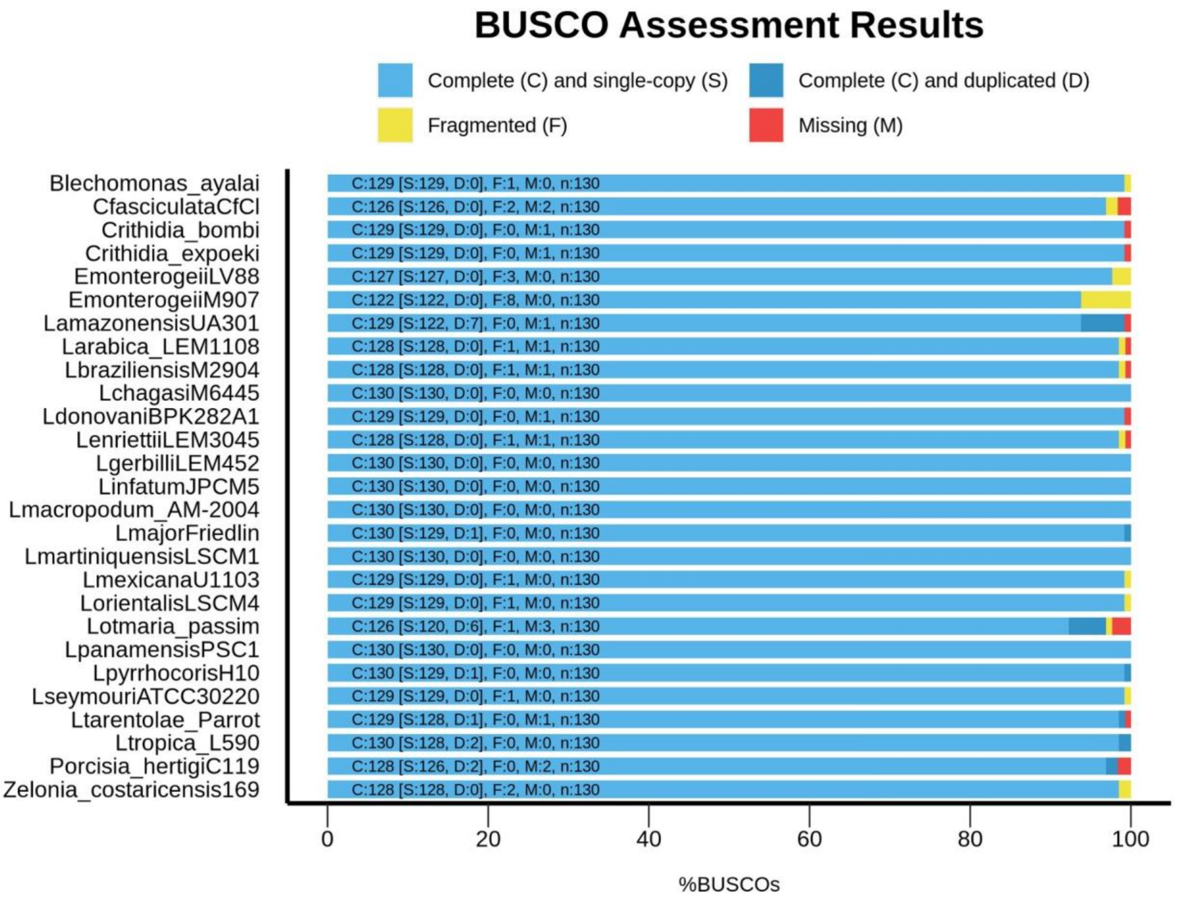

Figure S2. Genomic quality assessment of the 27 trypanosomatids genomes used in this study. The bar graph next to each species represents the BUSCO gene sections present or missing in each genome. Complete (C): fraction of single-copy, Duplicated (D): fraction of multiple-copy, Fragmented (F): fraction of genes with a partial sequence, Missing (M): fraction of genes not found in the genome.

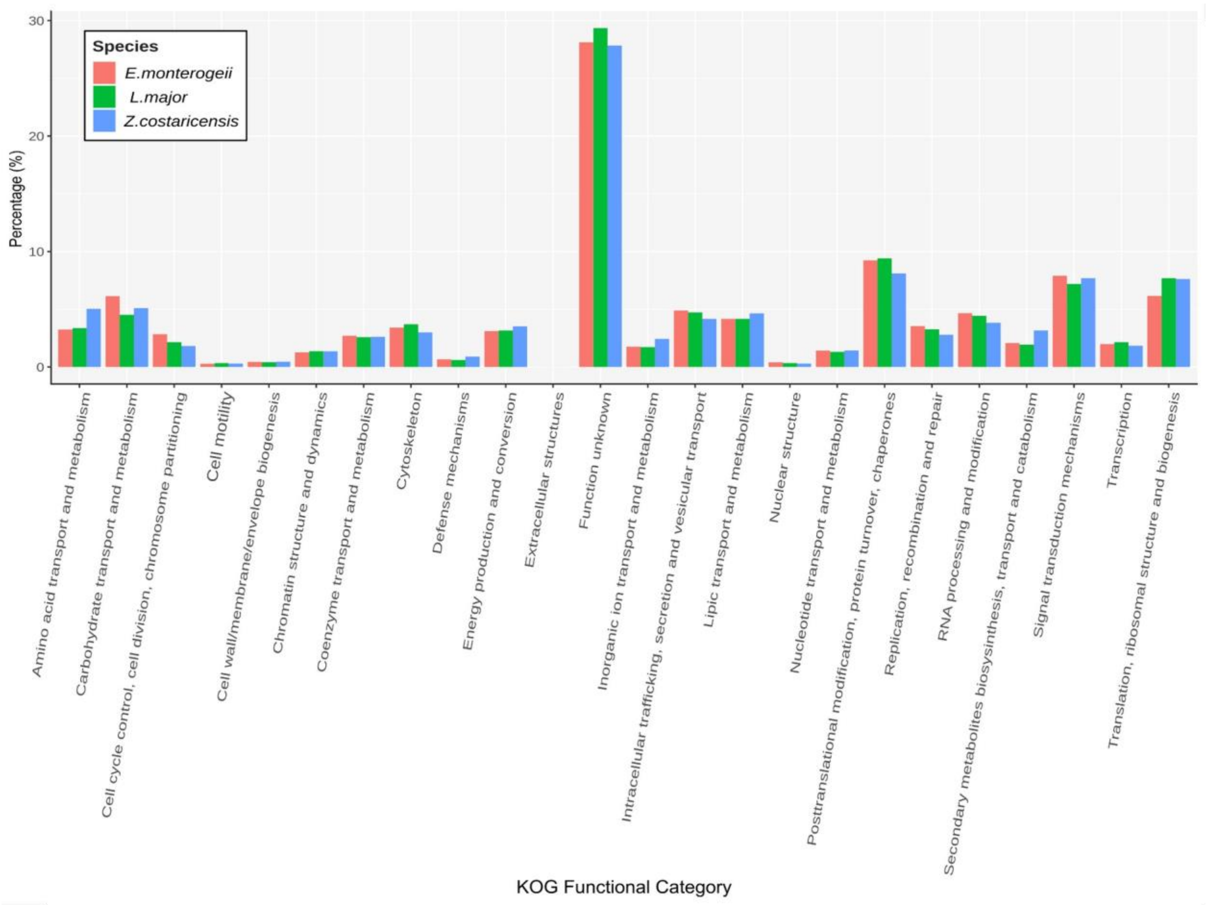

Figure S3. Histogram of functional classification of the genomes of the three species Leishmaniinae according to KOG functional groups. The comparison of the distribution of KOG functions for *L. major*, *E. monterogeii* and *Z. costaricensis* whole genome are shown.

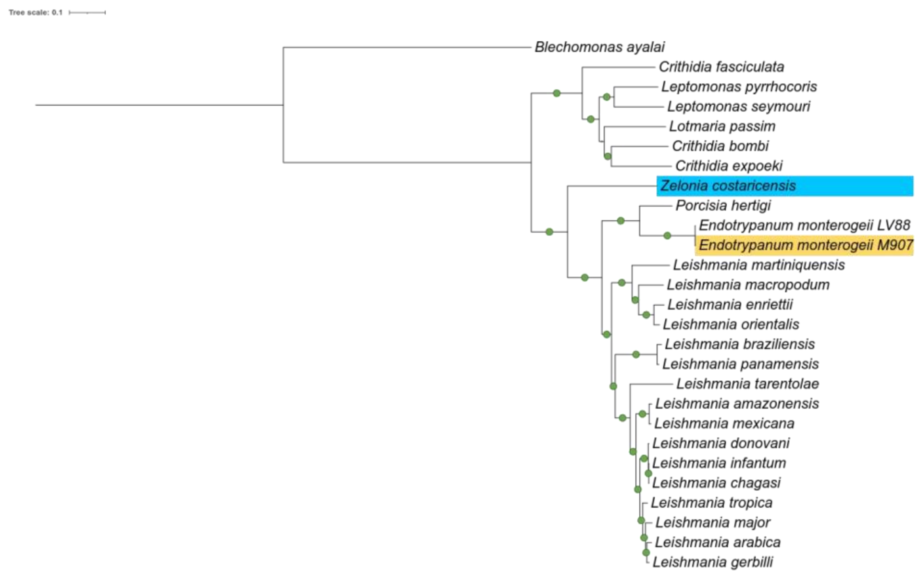

Figure S4.A. Phylogenomics analysis of subfamily Leishmaniinae based on the supermatrix method using 99 BUSCO conservative genes (JTT+Γ4 + F+BS). This topology tree was inferred with IQ-Tree2, and was used the JTT model (Jones-Taylor-Thornton) + Γ4 + F and with bootstrap=100%.

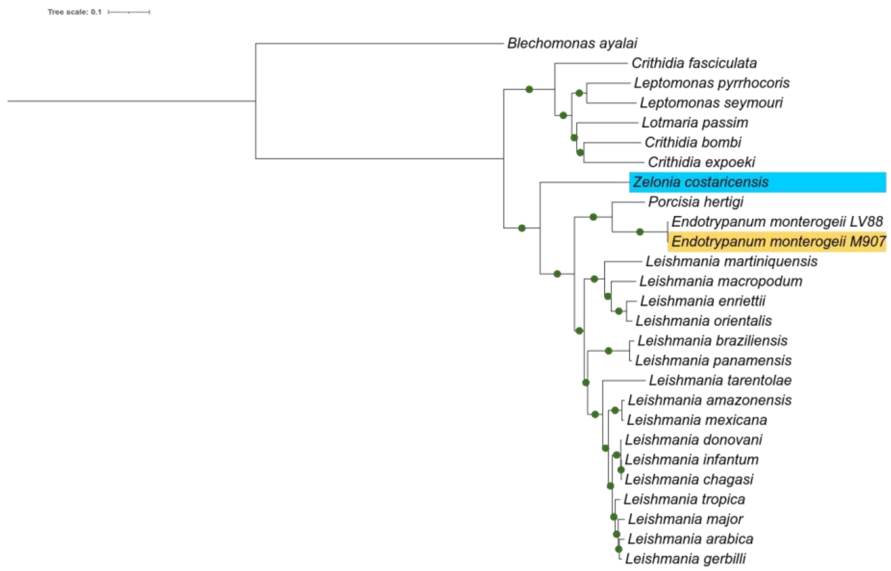

Figure S4.B. Phylogenomics analysis of subfamily Leishmaniinae based on the supermatrix method using 99 BUSCO conservative genes (JTT+Γ4 + F+UFBS). This topology tree was inferred with IQ-Tree2, and was used the JTT model + Γ4 + F and with Ultrafast bootstrap (UFBS)=100%.

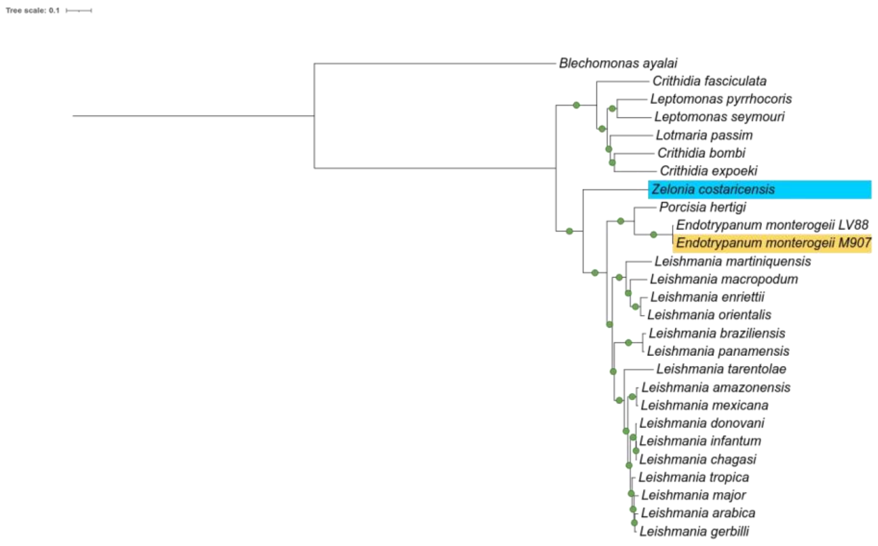

Figure S4.C. Phylogenomics relationships of subfamily Leishmaniinae based on the supermatrix method using 99 BUSCO conservative genes (PMSF+UFBS). This topology tree was inferred with IQ-Tree2, and was used the PMSF model and with Ultrafast bootstrap (UFBS)=100%.

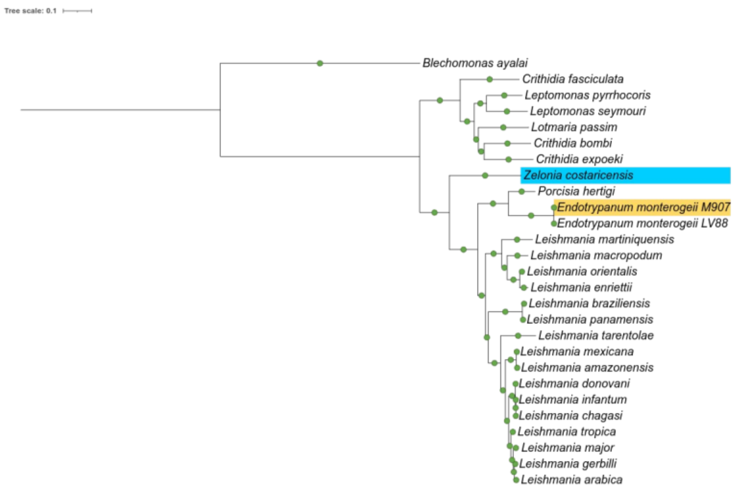

Figure S4.D. Phylogenomics relationships of subfamily Leishmaniinae based on the supermatrix method using 99 BUSCO conservative genes (Inference Bayesian). This topology tree was inferred with MrBayes, and support of branch PP=1

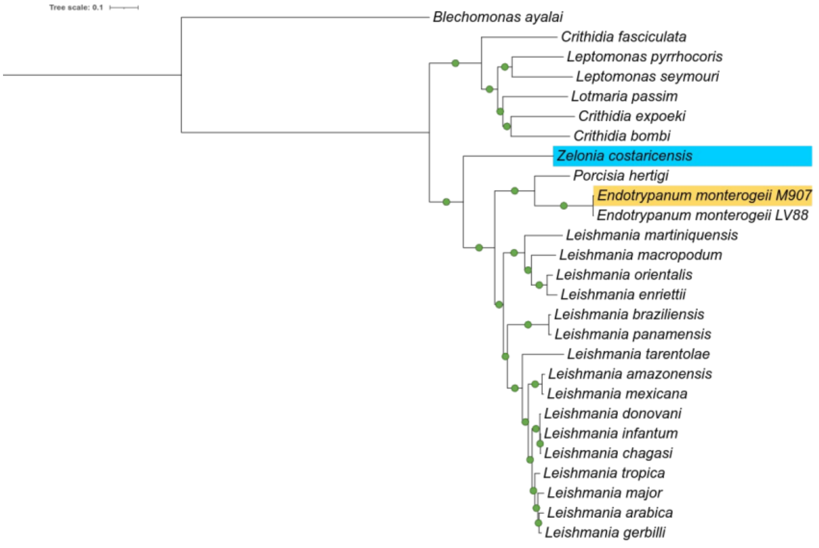

Figure S4.E. Phylogenomics relationships of subfamily Leishmaniinae based on supermatrix analyses (JTT+ Γ4 +F + UFBS) on 3384 single-copy genes. The topological structure was inferred by IQ-Tree2 using 3384 single-copy genes. The node's support was 100% using UFBS and JTT+ Γ4 +F model.

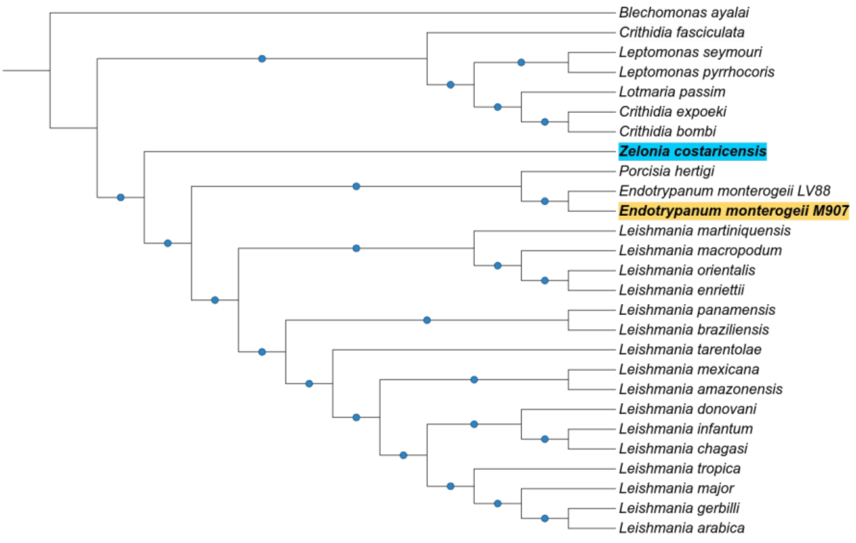

Figure S5. Phylogenomics relationships of subfamily Leishmaniinae based on supertree analyses. The supertree topological structure was inferred by ASTRAL using 3384 single-copy genes. The nodes from source trees (each gene tree) with bootstrap support below 70 were collapsed.

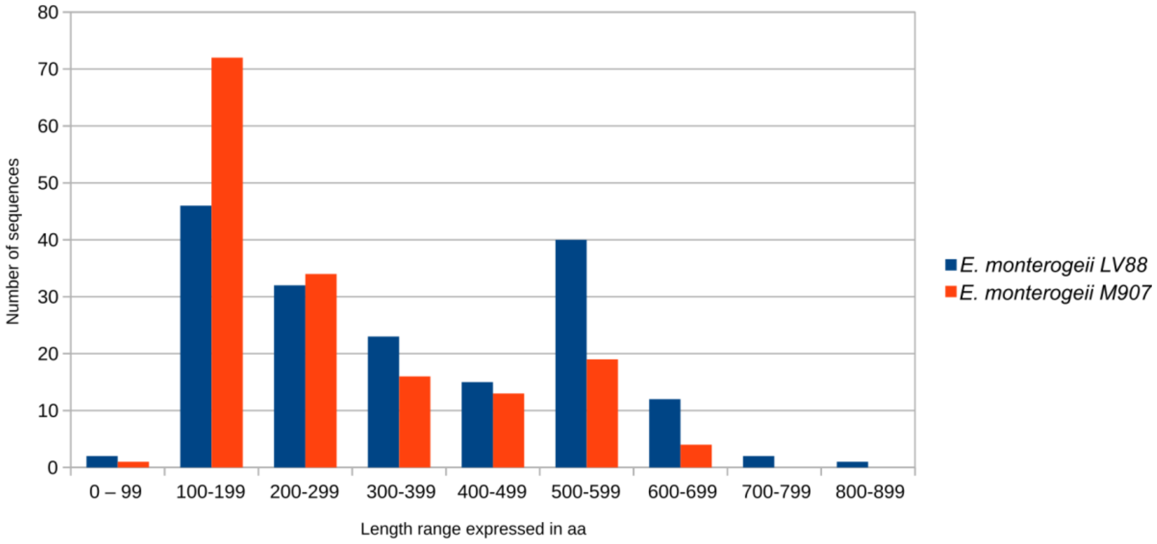

Figure S6. A bar graph indicating the comparison of the number of sequences between *E. monterogeii* LV88 (from TritrypDB) and *E. monterogeii* M907. The distribution is similar in both species, with a high number of copies in the range of 100-199 amino acids. Two genomes analyzed encoded a similar number of genes, with the genome of *E. monterogeii M907* encoding the lower (161) than of *E. monterogeii* LV88(171).

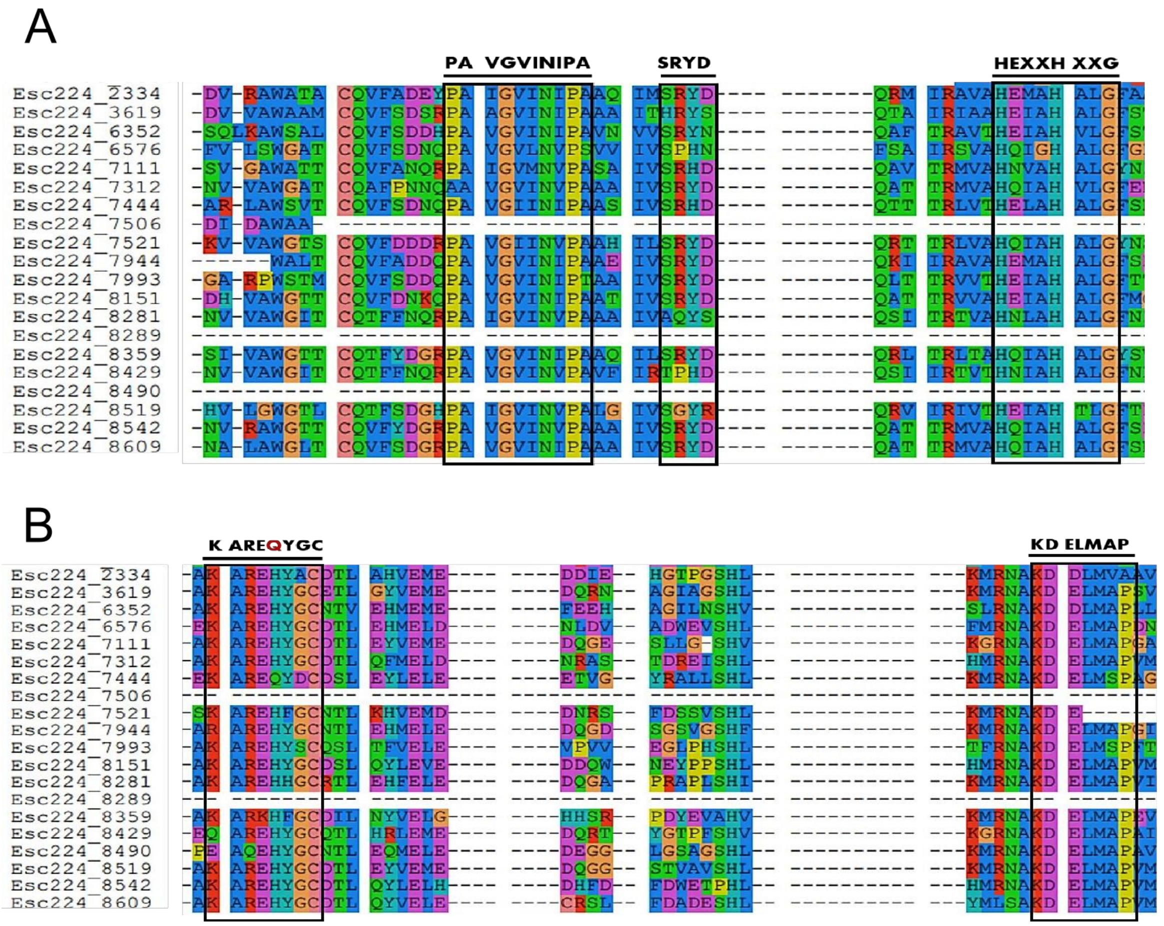

Figure S7. GP63 conserved motif in *E. monterogeii* M907. Alignments showing the conserved motif that include (A) PA-VGVINIPA, SRYD, HExxHxxG, and (B) KAREHYGC and KDELMAP, which have been seen in GP63 protein family from *L. braziliensis* [28].
